# Increasing the number of midwives is necessary but not sufficient: using global data to support the case for investment in both midwife availability and the enabling work environment in low- and middle-income countries

**DOI:** 10.1186/s12960-024-00925-w

**Published:** 2024-07-22

**Authors:** Andrea Nove, Martin Boyce, Sarah Neal, Caroline S. E. Homer, Tina Lavender, Zoë Matthews, Soo Downe

**Affiliations:** 1 Novametrics Ltd, 4 Cornhill Close, Duffield, Derby, DE56 4HQ United Kingdom; 2 Department of Social Statistics and Demography, University of Southampton, Southampton, United Kingdom; 3 Maternal, Child and Adolescent Health, Burnet Institute, Melbourne, VIC Australia; 4 Department of International Public Health, Centre for Childbirth, Women’s and Newborn Health, Liverpool School of Tropical Medicine, Liverpool, United Kingdom; 5 School of Nursing and Midwifery, University of Central Lancashire, Preston, United Kingdom

**Keywords:** Midwives, Midwifery, Maternal mortality, Neonatal mortality, Caesarean section, Model of care, Value based healthcare

## Abstract

**Background:**

Most countries are off-track to achieve global maternal and newborn health goals. Global stakeholders agree that investment in midwifery is an important element of the solution. During a global shortage of health workers, strategic decisions must be made about how to configure services to achieve the best possible outcomes with the available resources. This paper aims to assess the relationship between the strength of low- and middle-income countries’ (LMICs’) midwifery profession and key maternal and newborn health outcomes, and thus to prompt policy dialogue about service configuration.

**Methods:**

Using the most recent available data from publicly available global databases for the period 2000–2020, we conducted an ecological study to examine the association between the number of midwives per 10,000 population and: (i) maternal mortality, (ii) neonatal mortality, and (iii) caesarean birth rate in LMICs. We developed a composite measure of the strength of the midwifery profession, and examined its relationship with maternal mortality.

**Results:**

In LMICs (especially low-income countries), higher availability of midwives is associated with lower maternal and neonatal mortality. In upper-middle-income countries, higher availability of midwives is associated with caesarean birth rates close to 10–15%. However, some countries achieved good outcomes without increasing midwife availability, and some have increased midwife availability and not achieved good outcomes. Similarly, while stronger midwifery service structures are associated with greater reductions in maternal mortality, this is not true in every country.

**Conclusions:**

A complex web of health system factors and social determinants contribute to maternal and newborn health outcomes, but there is enough evidence from this and other studies to indicate that midwives can be a highly cost-effective element of national strategies to improve these outcomes.

**Supplementary Information:**

The online version contains supplementary material available at 10.1186/s12960-024-00925-w.

## Background

At current rates of progress, global targets for maternal and neonatal mortality reduction and increased coverage of essential maternal and newborn health services will not be met [1]. There is unprecedented alignment among key global stakeholders—such as the World Health Organization (WHO), the United Nations Population Fund (UNFPA), and bilateral partners—that investment in midwives and the midwifery model of care is a vital element of strategies to address shortcomings in the availability and quality of maternal and newborn health care [2–5].

The midwifery model promotes and advocates for a collaborative, human rights-based, respectful and woman-centred approach to care that respects women’s capacity to assume responsibility for their own health and avoids unnecessary clinical interventions during pregnancy, childbirth and the postpartum period, while ensuring skilled clinical care, including recognition and referral when problems arise [6]. Although this model can in theory be provided by any appropriately qualified health professional, midwives are unique in having expertise across the whole continuum of sexual, reproductive, maternal and newborn health (SRMNAH) care, and in subscribing to a woman-centred philosophy [7].

It is often assumed that midwives can only provide care for women at low risk of complications, but the 2014 Lancet Series on Midwifery provided evidence that all women can benefit, including those who additionally need medical intervention at specific points in time [8]. This has been expressed as “every woman needs a midwife, and some women need a doctor too” [9].

WHO recommends midwife-led care in settings with well-functioning midwifery programmes [10]. Midwife-led care can achieve comparable or better outcomes than other models of care, [11] often at a lower cost [12, 13]. Much of the evidence of the safety and benefits of care by midwives comes from high-income settings, but recently studies have emerged from low- and middle-income countries (LMICs). For example, a recent modelling study considered 88 LMICs and concluded that midwives who are educated and regulated according to global standards and who have an enabling work environment have the potential to avert two-thirds of the world’s maternal and neonatal deaths and stillbirths [14]. Another study found that the introduction of midwives to a hospital in Bangladesh led to improved quality of care for women and newborns [15]. A four-country study highlighted some universal enablers for the successful operation of midwife-led birthing centres [16].

Despite this body of evidence, maternity care provision in many countries continues to rely heavily on a risk-based bio-medical approach, which treats all pregnancies and births as potentially pathological. This is particularly acute in middle-income countries and in private sector settings, where rates of caesarean birth, for example, can reach over 50% [17]. There is some evidence that this trend is increasing in low-income countries too [18–20]. This approach can achieve low levels of maternal and neonatal mortality, but it raises questions about the longer-term impact of overmedicalization on the health and well-being of women and newborns and about cost-effectiveness [21]. It could also impact population safety if scarce human resources for health are not available for those in real need because they are conducting unnecessary interventions on those who are healthy. This is of particular concern in the context of a global shortage of doctors and other health workers, [22] such that the global need for health care—including SRMNAH care—cannot be met by the current workforce. Strategic decisions need to be made about how best to configure SRMNAH services with the available resources.

The impact of midwifery is constrained by midwife shortages and additionally by challenges such as inadequate education and training, restrictive scopes of practice, lack of an enabling work environment (i.e. one that supports the infrastructure, profession, and system-level integration needed for midwives to effectively practise to their full scope of work [23]), and gender-based discrimination [24, 25]. Despite these potential constraints some countries, including LMICs, have chosen to invest in midwives and others are planning to do so [26].

This paper aims to assess the relationship between a country’s midwife density (number of midwives per 10,000 population) and key maternal and newborn health indicators and the strength of its midwifery profession. As governments and health systems decide how best to deliver SRMNAH care with scarce resources, it is hoped that these results will prompt policy dialogue about how best to configure SRMNAH services.

Although the workforce challenges described above apply across the world, the impact is felt most in LMICs where maternal and newborn health outcomes are the poorest. For this reason, the analyses presented in this paper focus solely on LMICs, but the findings make it clear that LMICs should not be considered as a homogenous group in relation to the configuration of SRMNAH care services.

## Methods

This was an ecological study, during which we conducted descriptive analyses of national-level data from publicly available sources. Countries were eligible for inclusion if they were classed as LMICs by the World Bank in 2023 [27]. The World Bank classifies countries into one of four income groups based on their per capita gross national income: low, lower-middle, upper-middle, and high. We counted the first three of these four groups within our definition of LMIC.

We examined the association between midwife density (number of midwives per 10,000 population) and: (i) the maternal mortality ratio (MMR) (ii) the neonatal mortality rate (NMR), and (iii) the caesarean birth rate. Data sources and access dates for each of these indicators can be found in Additional file 1, Table A. Midwife density is reported by national ministries of health to WHO, using the National Health Workforce Accounts (NHWA) platform, based on the number of ‘midwifery personnel’ in the country, i.e. including both professional and associate professional midwives.

Countries were excluded from an analysis if there were missing data for at least one of the indicators under consideration. The countries included in each of the analyses in this paper are shown in Additional file 1, Table B.

For analyses with a single time point, the most recent available data were used for each country. For analyses involving change over time, the earliest and most recent data points were used within the specified timeframe for that analysis. All data points were from the period 2000 to 2020.

To examine the association between the strength of the midwifery profession and MMR reduction, we created a composite measure of the strength of Midwifery Service Structures (MSS) in the form of an index score that takes into account 11 separate factors:

1. Policy: is there a national policy that supports midwife-led care during pregnancy, childbirth and/or the postnatal period?
2. Licensing: is there a licensing system for midwives that requires continual professional development for relicensing?
3. Scope of practice: are midwives authorised to provide all seven basic emergency obstetric and neonatal care (BEmONC) signal functions [28] and all five of a list of modern contraceptives (injections, pills, intrauterine devices, emergency contraception, and implants)?
4. Legislation: is there legislation recognising midwifery as distinct from nursing?
5. Regulation: are there national regulation processes that are specific to midwives?
6. Professional association: is there a Professional Association specifically for midwives?
7. Postgraduate education: does the country offer postgraduate education in midwifery (MSc or PhD)?
8. Educators: % of midwifery educators who are themselves midwives
9. Leaders: number of midwives in leadership positions in the Ministry of health, regulatory authorities and health facilities
10. Availability of midwife-led care: number of midwife-led birthing centres
11. Midwife density: number of midwives per 10,000 population

Data sources for each item can be found in Additional file 1, Table A. Most of the data were collected in 2020 for inclusion in the *State of the World’s Midwifery* 2021 report, [24] but the policy data (item 1) were from a 2018–19 WHO survey; the midwife-led care data (item 10) were from a 2021 ICM survey, and the density data (item 11) were from the most recent available year in or after 2010. We allocated each country an MSS score between zero and five for each of the 11 factors, according to the scheme shown in Additional file 1, Table C. Each factor received equal weighting, so the maximum score for each country was 55. Countries were included in this analysis if data were available for at least ten of the eleven factors. A country with missing data for one factor received a score of zero for that factor, so countries with data for only ten factors may have been slightly underscored.

## Results

### Relationship between midwife density and mortality

**Fig. 1:**
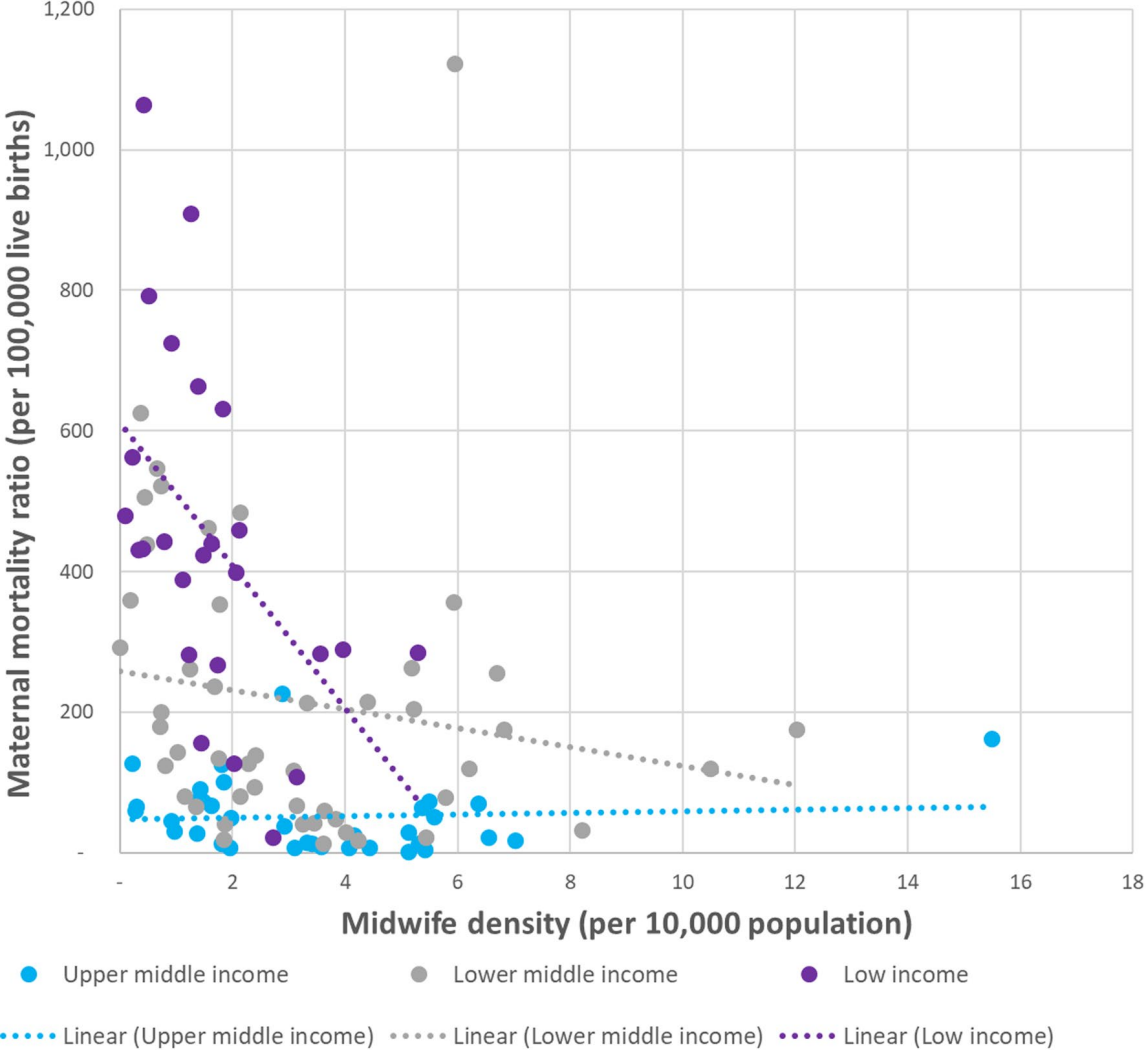
Relationship between maternal mortality ratio and midwife density for 108 low- and middle-income countries, by country income classification, most recent available year 2005–2020

**Fig. 2:**
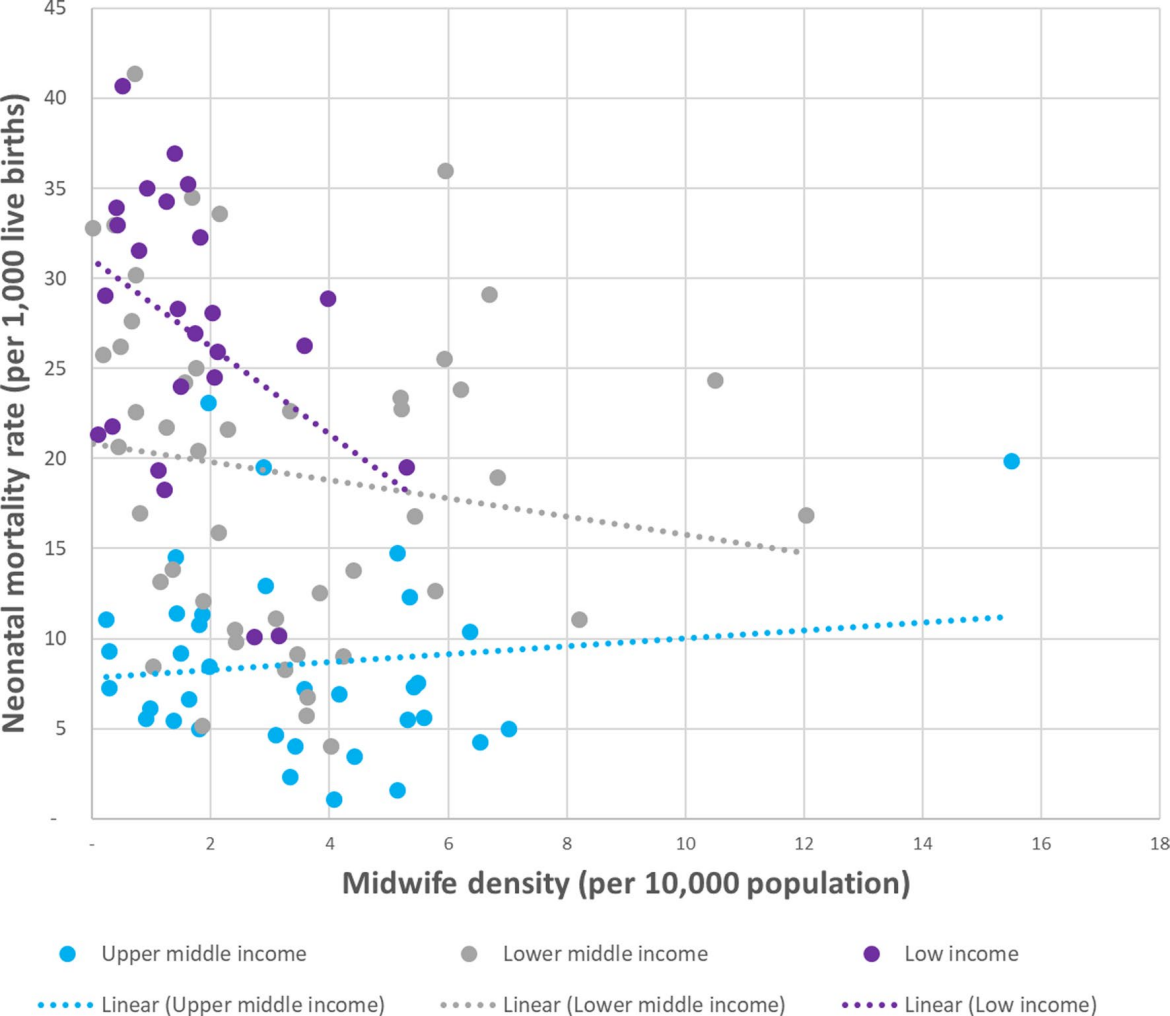
Relationship between neonatal mortality rate and midwife density for 108 low- and middle-income countries, by country income classification, most recent available year 2005–2020

Figures 1 and 2 plot midwife density against MMR and NMR respectively, with each point representing a country. Out of 133 LMICs, 112 reported midwife density in at least one year between 2005 and 2020. The chart shows the most recently reported density number, compared with the country’s MMR estimate for the same year. The three dotted lines represent the overall trend for low (purple), lower-middle (grey) and upper-middle-income (blue) countries respectively.

We excluded three countries (Marshall Islands, Palau and Tuvalu) due to having populations below 50,000, which makes it difficult to accurately measure mortality rates in a specific year. We also excluded Indonesia because in the years prior to 2020, Indonesia reported an extremely large midwife density, [24] yet in 2021 (after the cut-off point for inclusion in the charts) it acknowledged that the number of midwives had previously been significantly overestimated and revised its numbers downwards.

Figure 1 demonstrates that low- and lower-middle-income countries with a higher density of midwives tended to have a lower MMR and that this relationship was particularly strong for low-income countries. This was not true for upper-middle income countries, but this was entirely due to Botswana (represented by the blue dot on the right-hand side of the chart), which reported a very high midwife density and yet had a high MMR estimate, likely due at least in part to high rates of HIV in the country. If Botswana were to be excluded from the chart, the trend line for upper-middle-income countries would have the same orientation as for the other two country groupings. Figure 2 shows a similar, although a little less pronounced, pattern for the relationship between midwife density and NMR.

**Fig. 3:**
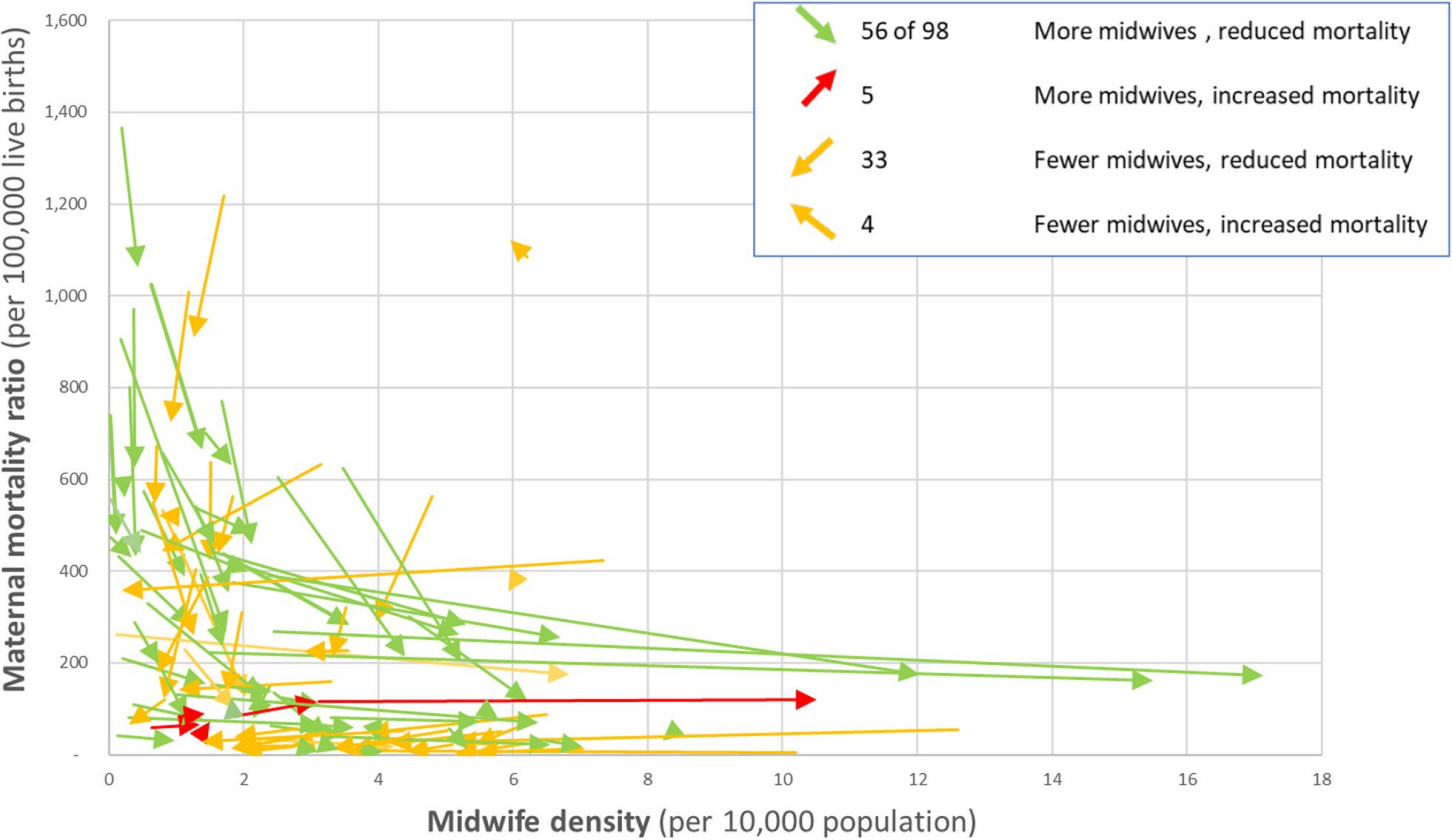
Relationship between midwife density change over time and maternal mortality reduction, for 98 low- and middle-income countries, 2000–2020

Few countries report midwife density every year, but 98 LMICs reported midwife density for at least two individual years between 2000 and 2020, which allows a limited analysis of time trends in the relationship between midwife density and mortality. In Fig. 3, each arrow represents a country, with the direction of the arrow showing the movement from earliest reported data to most recent within the 2000–2020 timeframe (for 7 countries, this date range was 5 years or less; 16 countries were between 6 and 10 years; 38 countries 11 to 15 years; and 37 countries 15–20 years). The arrows are colour-coded: for countries where midwife density increased and MMR reduced, arrows are green; for countries where both midwife density and MMR increased, arrows are red; other scenarios are yellow.

Figure 3 shows that most countries with high initial MMR estimates increased their midwife density thereafter, which was accompanied by a reduction in their MMR. Countries with a relatively low initial MMR demonstrated more mixed experiences. Some increased their midwife density and saw a simultaneous reduction in the MMR, but some achieved an MMR reduction without an increase in midwife density.

Overall, 56 of the 98 countries (57%) recorded an increase in midwife density and a reduction in MMR over the same time period, and a further four countries recorded a decrease in midwife density and an increased MMR. Only 5 countries increased their midwife density and recorded an increase in MMR over the same period (Belize, Jamaica, Kiribati, Federated States of Micronesia (FSM) and Viet Nam). Kiribati and FSM have small and dispersed populations, so an increased midwife density could be achieved by adding only a very small number of midwives, who would not necessarily be accessible to a large proportion of the population, e.g. if based on a single island.

The remaining 33 countries (34%) achieved a reduced MMR while also seeing a reduction in midwife density (NB this does not necessarily mean fewer midwives—rather that midwife numbers were not keeping pace with population growth). Of these 33 countries, half (*n* = 17) are middle-income countries with a highly medicalized system (e.g. Belarus, Ecuador, Russia, Tunisia, Turkmenistan, Ukraine). It is also notable that nearly all of the remaining countries in this group had a ‘starting’ MMR above 300 (e.g. Central African Republic, Guinea-Bissau, Haiti, Myanmar, Pakistan, Tanzania), indicating that MMR reduction could have been achieved in a number of different ways.

### Relationship between midwife density and caesarean birth rate

WHO does not recommend a specific caesarean birth rate, but has noted that as the rate rises towards 10% maternal and neonatal mortality decrease, whereas rates above 10% are not associated with mortality reduction [29]. As a general rule, a rate of below 10% is considered too low, and a rate of above 15% too high.

**Fig. 4:**
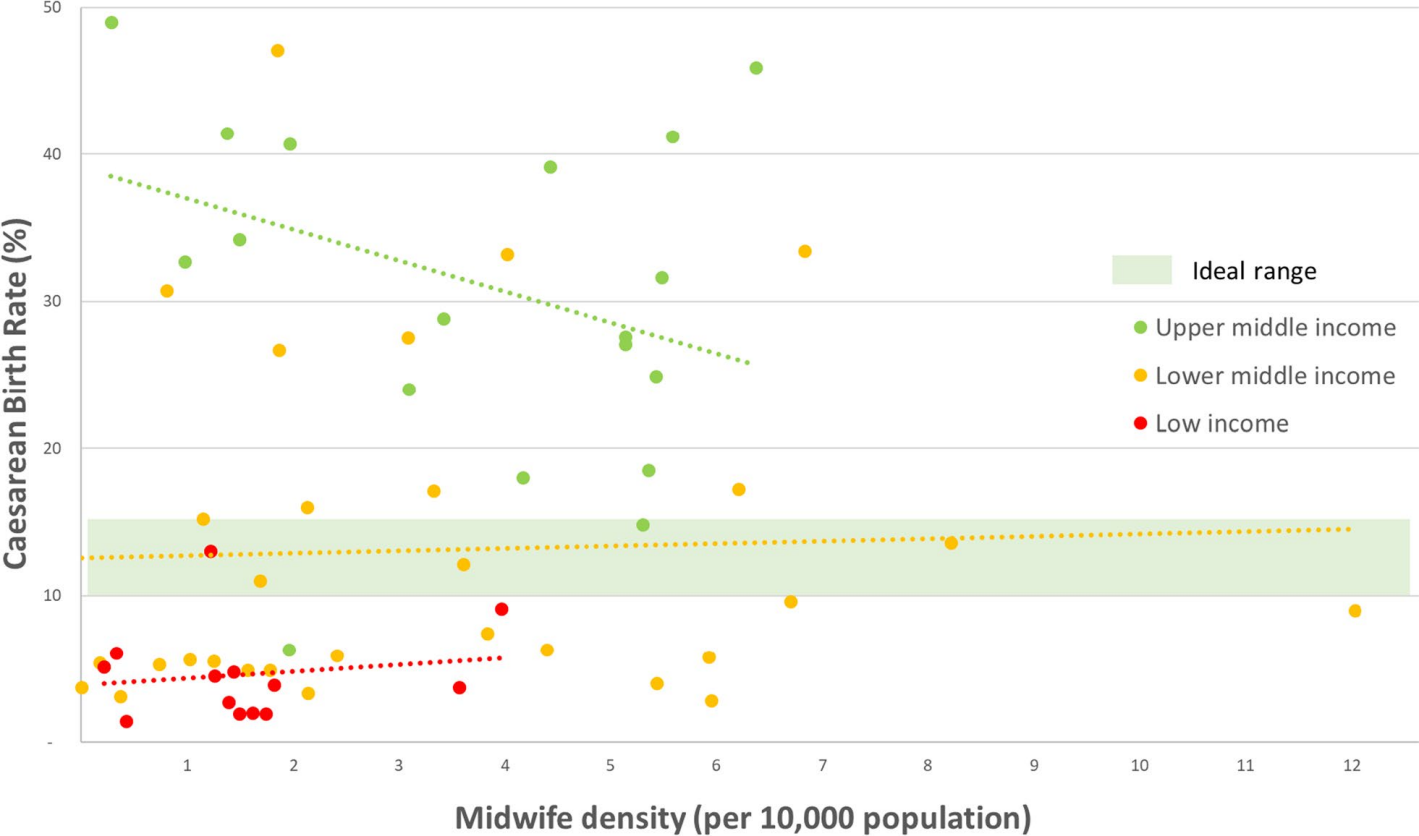
Relationship between caesarean birth rate and midwife density for 62 low- and middle-income countries, 2010–2020

We examined the caesarean birth rates reported by WHO for 62 LMICs for the most recent year between 2010 and 2020, and for each country plotted this against the midwife density for the closest available year, excluding any countries for which the two data points were more than 5 years apart. Figure 4 shows that almost all upper-middle income countries (shown in green) reported a caesarean birth rate above 10% (the exception being Turkmenistan), but there was a tendency for countries with a higher midwife density to have lower caesarean birth rates. Conversely, almost all low-income countries had rates below 10% (the exception being Rwanda), but those with higher midwife densities were somewhat more likely to be closer to the ideal range.

### Relationship between midwifery service structures and MMR reduction

**Fig. 5:**
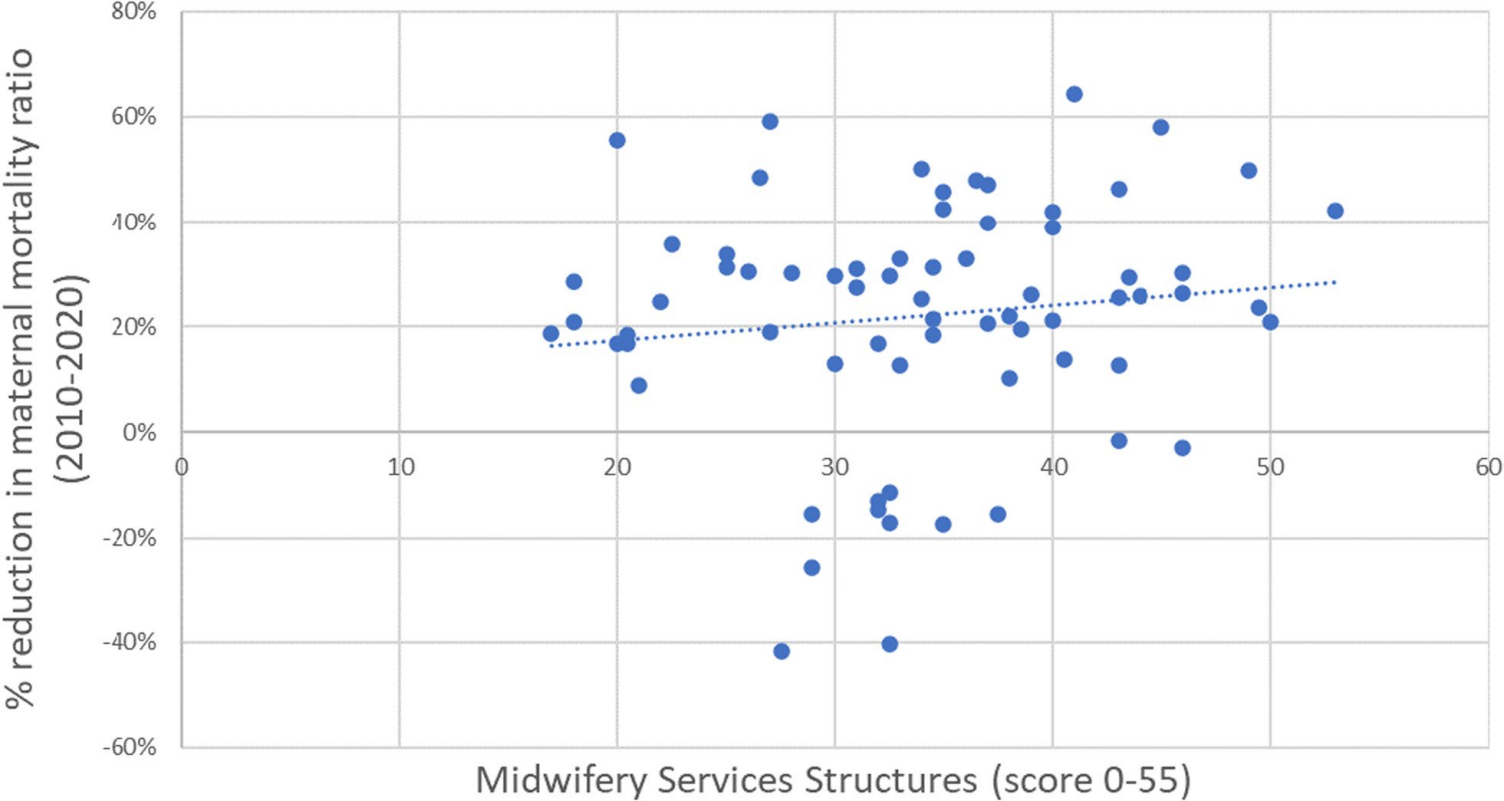
Relationship between Midwifery Service Structures score and maternal mortality reduction in 70 LMICs, 2010–2020. A negative MMR reduction indicates increased maternal mortality over time

Each dot in Fig. 5 represents one of the 70 countries with sufficient data to calculate an MSS index score. It shows that countries with stronger midwifery structures tended to achieve greater MMR reductions between 2010 and 2020. However, it was by no means a perfect correlation—some countries with weaker structures achieved relatively good mortality reduction, and some with stronger structures did not.

## Discussion

The analyses presented in this paper show that, in LMICs (and low-income countries in particular), higher availability of midwives is associated with lower maternal and neonatal mortality. In low- and upper-middle-income countries, higher availability of midwives is associated with caesarean birth rates closer to the ideal range of 10–15%. However, these general patterns are not universally true—some countries have achieved good outcomes without increasing midwife availability, and some have increased midwife availability and not achieved good outcomes. Similarly, we show in this paper that stronger midwifery service structures are associated with greater reductions in maternal mortality, but this is not true in every country.

The existence of exceptions to these general patterns underscores the complexity of the web of health system factors and social determinants that contribute to maternal and newborn health outcomes. It would be over-simplistic to suggest that the association between midwife density and health outcomes is necessarily a direct and causal one. It is possible that one or more completely different factors are responsible for the patterns observed in the health outcomes data. However, in the context of (i) our finding that hardly any countries recorded an increase in maternal mortality with more midwives, and (ii) a wealth of previous research showing the impact and potential impact of midwives, the hypothesis that investment in midwives contributes to improved outcomes is plausible and worthy of closer examination. It would be highly complex to design and conduct a multi-country quantitative study to provide a definitive answer to the question of causality. Additional insights could, however, be drawn from high-quality mixed-methods studies based on analysis of policy and health systems development data and documentation from a range of individual countries. Midwives can only fulfil their potential to improve outcomes if they are supported and enabled to do so. [30] The midwifery service structures analysis presented in this paper highlights the need for a focus on the policy context, education, regulation and strengthening the profession as well as on midwife numbers. Furthermore, LMICs that have invested successfully in midwives tend first to pay attention to the infrastructure within which the midwives work. [26] Without attention to the work environment, efforts to build the midwifery workforce risk being wasted due to poor midwife recruitment and retention. Similarly, to be fully effective, midwives must work within a supportive, mutually respectful and multidisciplinary team that is collectively able to ensure that all women and newborns receive the appropriate care at the appropriate time from the appropriate clinician(s). [24] It is likely that some of the exceptions to the general patterns observed in this study occurred because insufficient attention had been paid to ensuring an enabling work environment for the midwifery workforce.

The data make it clear that reduced maternal and neonatal mortality can be achieved either with or without investment in midwives. Mortality is without doubt an important metric, not least because death is arguably the worst outcome of a pregnancy, and also because it is relatively straightforward to measure. However, it is not the only outcome that matters. The benefits of high-quality midwifery go well beyond saving lives. For example, evidence shows that midwives can: encourage health facilities to use evidence-informed care practices, [15] improve health equity, [31] and improve psychological safety. [32] Furthermore, studies have shown midwife-led care is capable of yielding results that are at least as good as other models of care at a lower cost. [12, 13, 33, 34]

Additionally, the analysis in this paper relating to the caesarean birth rate suggests that midwives may have a protective effect against both the over- and the under-medicalization of childbirth that has been observed in many countries. [17–20] Countries with low coverage of caesarean births should be thinking about how to ensure access to this life-saving intervention for those who need it, while simultaneously thinking about how to avoid over-use. Midwives with a philosophical commitment and a clinical skillset to support physiological birth when this is clinically appropriate, and the competencies to stabilise and refer when medical intervention is required, are the obvious health professionals to help ensure this balance is achieved.

As noted earlier, the tendency for countries at all income levels to prioritise a risk-based biomedical approach to maternity care has improved outcomes for some and compromised the safety of others. Deployment of scarce human resources for health towards medically unnecessary intervention for healthy women harms other (often poorer) women who need intervention but cannot access it. Global targets about skilled birth attendance have resulted in strategies to encourage more women to give birth in health facilities. However, facility birth does not automatically result in better outcomes. It is not sufficient just to encourage women to give birth in facilities without at the same time investing in the workforce and infrastructure. A recent study in Ghana concluded that a higher rate of facility birth results in lower mortality only if the facilities are capable both of providing emergency obstetric and newborn care and of safeguarding uncomplicated births. [35] The midwifery model of care is the obvious way for this to be fully achieved. Our analysis suggests that increasing the availability of midwives with expertise in optimising physiological processes and identifying and treating or referring complications, could address both under- and over-treatment in maternity care in all settings.

Even when midwives’ positive impact on health outcomes is recognised, governments can be reluctant to increase their numbers, seeing health workers as a cost rather than an investment. There is, however, increasing evidence that investment in health workers not only improves health but also has multiplier effects on the broader economy [36] and is vital for improving the retention and resilience of health workers. [37] Investing in a female-dominated profession such as midwifery may also bring about positive impacts on women’s empowerment.

### Limitations

Midwife density data in global databases is not always complete and not always accurate. [38] It is therefore possible that some of the data presented in this paper are incorrect. It is vital that countries invest in health workforce data systems that permit robust and reliable analysis such as that attempted in this paper. In relation to midwives in particular, these data systems must be able to make a clear distinction between nurses and midwives (especially in countries that have a dual nursing and midwifery qualification) and between professional and associate professional midwives. For midwives and all other health workers, data systems should be able to monitor key indicators such as contracted hours, work location and work function, to avoid over- or under-estimation of availability of clinical workers and to enable sub-national analyses.

Similarly, the MMR and NMR figures used in these analyses are modelled estimates with wide confidence intervals. They are often quite different from national estimates produced using other methods. The level of uncertainty surrounding these estimates can make it difficult to measure change over time.

The mortality analyses shown in this paper would ideally have included stillbirth as well as maternal and neonatal mortality. However, the available data on stillbirth are scarce and vary little between countries, which calls into question their accuracy. We therefore decided not to include this important health outcome measure.

Ecological studies such as this one use a country as the unit of analysis, whereas some of the variables used in the analysis (e.g. mortality) apply at the level of an individual person. Neither do the analyses take into account the effects of potential confounders. For these reasons, we cannot assume a causal relationship between midwife density and mortality/caesarean birth, especially not at the level of the individual woman.

The midwifery service structures index used in this paper was developed specifically for this study and is not yet tried and tested. The index might be improved by applying an evidence-informed weighting scheme to emphasise particularly influential factors. The validation of the index scores and the development of a weighting scheme would require additional expert consultation, which was beyond the scope of this study. However, as the index is built on a range of factors that are recognized as important for a strong midwifery profession, we believe that it provides useful insights and warrants further development in the future.

## Conclusions

There is a growing international appreciation that important outcomes of maternity care are not limited to mortality and morbidity. While the introduction or strengthening of a range of models of maternity care has been associated with reductions in death and serious harm for mothers and newborns, there has been less progress in the implementation of respectful care, or in recognition of the psychological and social importance of a good birth experience, for the mother, her partner, and her family, in the short and longer term. Midwives are now recognised as skilled and able to provide both clinical and psychosocial care across the whole maternity continuum. The introduction of midwifery is on the agenda of an increasing number of global agencies and national governments. Our analyses have shown that increasing the availability of midwives tends to be associated with reductions in adverse outcome for mother and baby, and in rates of caesarean birth that are closer to the recommended level. However, this association is not always evident. Further analyses using our midwifery services structure index suggest that just growing midwife numbers is not enough. Strengthening the infrastructure in terms of policy, licencing, education, scope of practice, regulation, leadership and availability of staff is likely to catalyse higher benefits than simply having more midwives working in existing maternity care systems. We encourage those who are currently considering midwifery as a solution to the phenomenon of ‘too little too late, too much too soon’ to take these findings into account.

### Supplementary Information

Supplementary Material 1.

## Data Availability

All the results presented in this paper are based on secondary analysis of publicly available data, which are cited as appropriate and therefore, accessible to the interested reader.

## Abbreviations

BEmONC: Basic emergency obstetric and neonatal care
FSM: Federated States of Micronesia
LMICs: Low- and middle-income countries
MMR: Maternal mortality ratio
MSS: Midwifery service structures
NHWA: National Health Workforce Accounts
NMR: Neonatal mortality rate
SRMNAH: Sexual, reproductive, maternal, newborn and adolescent health
UNFPA: United Nations Population Fund
WHO: World Health Organization
