# Supplementary material for: Increasing the number of midwives is necessary but not sufficient: using global data to support the case for investment in both midwife availability and the enabling work environment in low- and middle-income countries
Source: Hum Resour Health. 2024 Jul 22;22:54. doi: 10.1186/s12960-024-00925-w (PMC11264417; doi:10.1186/s12960-024-00925-w)
Supplement: Supplementary file 1 — Supplementary Material 1. [file 12960_2024_925_MOESM1_ESM.docx]

**Additional file 1**

**Table A: Data sources and access dates**

| **Indicator** | **Data source** | **URL** | **Date accessed** |
| --- | --- | --- | --- |
| Midwifery personnel total density (per 10,000 population) | WHO National Health Workforce Accounts | <https://apps.who.int/nhwaportal/Home/Index> | 2 June 2023 |
| Maternal mortality ratio (per 100,000 live births) | WHO Trends in maternal mortality 2000 to 2020 | <https://www.who.int/publications/i/item/9789240068759> | 1 June 2023 |
| Neonatal mortality rate (per 1,000 live births) | UNICEF | <https://data.unicef.org/topic/child-survival/neonatal-mortality/> | 1 June 2023 |
| Births by caesarean section, % | WHO Global Health Observatory | <https://www.who.int/data/gho/data/indicators/indicator-details/GHO/births-by-caesarean-section-(-)> | 18 Sept 2023 |
| Place of delivery: Health facility, % | StatCompiler, except for Viet Nam 2021 for which the data were taken from the MICS report | [www.StatCompiler.com](http://www.StatCompiler.com) | 3 Aug 2023 |
| Existence of a policy that supports midwife-led care during pregnancy, childbirth and postnatal period? | 2018-2019 WHO SRMNCAH policy survey | <https://platform.who.int/data/maternal-newborn-child-adolescent-ageing/national-policies> | 1 Feb 2021 |
| Midwife licensing system | International Confederation of Midwives Global Midwives’ Hub | <https://www.globalmidwiveshub.org/> | 16 Feb 2021 |
| Midwives’ scope of practice (BEmONC functions and contraceptive products) |  |  |  |
| Legislation recognising midwifery as distinct from nursing |  |  |  |
| Regulatory processes that are specific to midwives |  |  |  |
| Professional association specifically for midwives |  |  |  |
| Postgraduate education in midwifery |  |  |  |
| % of midwife educators who are themselves midwives |  |  |  |
| Number of midwife leaders (in national and sub-national ministry of health, regulatory authorities, health facilities) |  |  |  |
| Existence of midwife-led birthing centres | Nove et al 2023 | <https://www.sciencedirect.com/science/article/pii/S0266613823001201?via%3Dihub> | 11 Aug 2023 |

**Table B: Which countries were included in each analysis**

| **Country** | **Fig. 1** | **Fig. 2** | **Fig. 3** | **Fig. 4** | **Fig. 5** |
| --- | --- | --- | --- | --- | --- |
| Afghanistan | YES | YES | YES | YES | YES |
| Albania | YES | YES | YES |  |  |
| Algeria | YES | YES | YES | YES | YES |
| Angola | YES | YES |  | YES |  |
| Argentina | YES | YES |  |  | YES |
| Armenia | YES | YES | YES | YES |  |
| Azerbaijan | YES | YES | YES | YES |  |
| Bangladesh | YES | YES | YES | YES | YES |
| Belarus | YES | YES | YES | YES |  |
| Belize | YES | YES | YES | YES |  |
| Benin | YES | YES | YES | YES | YES |
| Bhutan |  |  |  |  | YES |
| Bolivia (Plurinational State of) | YES | YES | YES | YES | YES |
| Bosnia and Herzegovina | YES | YES | YES | YES |  |
| Botswana | YES | YES | YES |  |  |
| Brazil | YES | YES |  | YES | YES |
| Bulgaria | YES | YES | YES | YES | YES |
| Burkina Faso | YES | YES | YES | YES | YES |
| Burundi | YES | YES | YES |  | YES |
| Cabo Verde |  |  |  |  |  |
| Cambodia | YES | YES | YES | YES |  |
| Cameroon | YES | YES | YES |  | YES |
| Central African Republic (the) | YES | YES | YES | YES |  |
| Chad | YES | YES | YES | YES | YES |
| China |  |  |  |  |  |
| Colombia |  |  |  |  |  |
| Comoros | YES | YES | YES | YES | YES |
| Congo | YES | YES | YES | YES | YES |
| Congo, Democratic Republic of the | YES | YES | YES | YES |  |
| Costa Rica |  |  |  |  |  |
| Côte d'Ivoire | YES | YES | YES | YES | YES |
| Cuba |  |  |  |  |  |
| Djibouti | YES | YES | YES | YES |  |
| Dominica |  |  |  |  |  |
| Dominican Republic |  |  |  |  |  |
| Ecuador | YES | YES | YES | YES | YES |
| Egypt |  |  |  |  |  |
| El Salvador |  |  |  |  |  |
| Equatorial Guinea |  |  |  |  |  |
| Eritrea | YES | YES | YES |  | YES |
| Eswatini |  |  |  |  | YES |
| Ethiopia | YES | YES | YES | YES | YES |
| Fiji | YES | YES |  |  |  |
| Gabon | YES | YES | YES |  | YES |
| Gambia | YES | YES | YES |  | YES |
| Georgia | YES | YES | YES | YES |  |
| Ghana | YES | YES | YES |  | YES |
| Grenada |  |  |  |  |  |
| Guatemala |  |  |  |  |  |
| Guinea | YES | YES | YES | YES | YES |
| Guinea-Bissau | YES | YES | YES |  | YES |
| Haiti | YES | YES | YES | YES | YES |
| Honduras | YES | YES | YES |  |  |
| India | YES | YES | YES | YES | YES |
| Indonesia |  |  | YES |  | YES |
| Iran (Islamic Republic of) | YES | YES | YES |  | YES |
| Iraq | YES | YES | YES |  | YES |
| Jamaica | YES | YES | YES |  |  |
| Jordan | YES | YES | YES |  |  |
| Kazakhstan | YES | YES | YES | YES |  |
| Kenya | YES | YES |  |  | YES |
| Kiribati | YES | YES | YES |  |  |
| Korea, Democratic People's Republic of | YES | YES | YES |  |  |
| Kyrgyzstan | YES | YES | YES | YES | YES |
| Lao People's Democratic Republic | YES | YES | YES |  |  |
| Lebanon | YES | YES | YES | YES | YES |
| Lesotho |  |  |  |  |  |
| Liberia | YES | YES | YES | YES | YES |
| Libya | YES | YES |  |  | YES |
| Madagascar | YES | YES | YES | YES | YES |
| Malawi | YES | YES | YES | YES | YES |
| Malaysia | YES | YES | YES |  |  |
| Maldives | YES | YES |  | YES |  |
| Mali | YES | YES | YES | YES | YES |
| Marshall Islands (Republic of) |  |  |  |  |  |
| Mauritania | YES | YES | YES | YES | YES |
| Mauritius |  |  |  |  |  |
| Mexico | YES | YES |  | YES | YES |
| Micronesia (Federated States of) | YES | YES | YES |  |  |
| Moldova (Republic of) | YES | YES | YES |  |  |
| Mongolia | YES | YES | YES |  | YES |
| Montenegro | YES | YES | YES |  |  |
| Morocco | YES | YES | YES | YES | YES |
| Mozambique | YES | YES | YES |  |  |
| Myanmar | YES | YES | YES | YES | YES |
| Namibia |  |  |  |  | YES |
| Nepal | YES | YES | YES | YES | YES |
| Nicaragua |  |  |  |  |  |
| Niger | YES | YES | YES |  |  |
| Nigeria | YES | YES | YES | YES |  |
| North Macedonia | YES | YES | YES | YES |  |
| Pakistan | YES | YES | YES |  |  |
| Palau |  |  |  |  |  |
| Papua New Guinea | YES | YES | YES |  |  |
| Paraguay | YES | YES | YES | YES | YES |
| Peru | YES | YES | YES | YES | YES |
| Philippines | YES | YES | YES |  | YES |
| Russian Federation | YES | YES | YES |  |  |
| Rwanda | YES | YES | YES | YES | YES |
| Saint Lucia | YES | YES |  | YES |  |
| Saint Vincent and the Grenadines |  |  |  |  |  |
| Samoa | YES | YES | YES |  |  |
| Sao Tome and Principe | YES | YES | YES | YES |  |
| Senegal | YES | YES | YES | YES | YES |
| Serbia | YES | YES | YES | YES | YES |
| Sierra Leone | YES | YES | YES |  | YES |
| Solomon Islands | YES | YES | YES | YES | YES |
| Somalia | YES | YES |  |  | YES |
| South Africa | YES | YES |  |  |  |
| South Sudan |  |  |  |  | YES |
| Sri Lanka | YES | YES | YES | YES |  |
| Sudan | YES | YES | YES | YES | YES |
| Suriname | YES | YES | YES |  | YES |
| Syrian Arab Republic | YES | YES | YES |  | YES |
| Tajikistan | YES | YES | YES | YES | YES |
| Tanzania, United Republic of | YES | YES | YES |  |  |
| Thailand | YES | YES | YES | YES | YES |
| Timor-Leste | YES | YES | YES |  | YES |
| Togo | YES | YES | YES |  | YES |
| Tonga | YES | YES | YES |  |  |
| Tunisia | YES | YES | YES | YES | YES |
| Türkiye | YES | YES | YES |  | YES |
| Turkmenistan | YES | YES | YES | YES |  |
| Tuvalu |  |  |  |  |  |
| Uganda | YES | YES | YES |  | YES |
| Ukraine | YES | YES | YES | YES |  |
| Uzbekistan | YES | YES | YES | YES |  |
| Vanuatu | YES | YES | YES |  | YES |
| Venezuela (Bolivarian Republic of) |  |  |  |  |  |
| Viet Nam | YES | YES | YES | YES | YES |
| Yemen | YES | YES | YES | YES | YES |
| Zambia | YES | YES | YES |  | YES |
| Zimbabwe | YES | YES | YES | YES | YES |

**Table C: Midwifery Services Structures scoring**

| **Factor** | **Description** | **Option** | **Score** |
| --- | --- | --- | --- |
| 1 | **Policy:** Is there a national policy that supports midwife-led care during pregnancy, childbirth and postnatal period? | YES, there is a national policy for mother & newborn, for all 3: pregnancy, childbirth and postnatal period | 5 |
|  |  | for each ‘mother & newborn’ | 1.5 ea. |
|  |  | For each ‘mother only’ | 1.0 ea. |
|  |  | No | 0 |
| 2 | **Licensing:** Is there a licensing system for midwives that requires continual professional development for relicensing? | Continued professional development (CPD) is a requirement for relicensing | 5 |
|  |  | There is periodic relicensing | 4 |
|  |  | Licensing is compulsory | 2 |
|  |  | Licensing system is not compulsory | 1 |
|  |  | No licensing system | 0 |
| 3 | **Scope of Practice:** Are midwives authorised to provide all seven BEmONC signal functions and all five contraceptive products? | Each YES scores  *Then deduct 1 point from total score, so maximum is 5* | 0.5 ea. |
| 4 | **Legislation:** Is their legislation recognising midwifery as distinct from nursing? | Yes | 5 |
|  |  | No | 0 |
| 5 | **Regulation:** Are there national regulation processes that are specific to midwives? | Separate regulation and separate regulatory authority for midwives | 5 |
|  |  | Separate regulation, but the regulatory authority also regulates other health professionals | 4 |
|  |  | Same regulations and regulatory authority as other health professionals | 2 |
|  |  | No regulation | 0 |
| 6 | **Professional Association:** Is there a Professional Association just for midwives? | Professional Association just for midwives | 5 |
|  |  | Professional Association open to midwives and other health professionals | 3 |
|  |  | No Professional Association for midwives | 0 |
| 7 | **Postgraduate education:** Does the country offer postgraduate education in midwifery (MSc or PhD)? | Doctorate-level midwifery education available in country | 5 |
|  |  | Masters-level midwifery education available in country | 3 |
|  |  | No postgraduate education in midwifery available in country | 0 |
| 8 | **Educators:** Are at least 50% of midwifery educators themselves midwives? | More than 50% of midwife educators are midwives themselves | 5 |
|  |  | Between 25% and 50% of midwife educators are midwives themselves | 3 |
|  |  | More than 0% and less than 25% of midwife educators are midwives themselves | 1 |
|  |  | No midwife educators are midwives themselves | 0 |
| 9 | **Leaders:** Are there midwives in leadership positions in the Ministry of Health, Regulatory Authorities and Health Facilities? | There is at least one midwife leader in the National Ministry of Health, sub-national MoH offices and in regulatory authorities | 5 |
|  |  | There is at least one midwife leader in the National Ministry of Health, but not anywhere else | 4 |
|  |  | There is at least one midwife leader in sub-national Ministry of Health offices, but not anywhere else | 3 |
|  |  | There is at least one midwife leader in the regulatory authorities, but not anywhere else | 2 |
|  |  | There is at least one midwife leader in a health facility, but not anywhere else | 1 |
|  |  | There are no midwives in leadership positions in country | 0 |
| 10 | **MLBCs:** Are there midwife-led birthing centres (MLBCs) in country? | There are ten or more MLBCs in country | 5 |
|  |  | There are one or more midwife-led centres or units, but none of them provide childbirth care | 4 |
|  |  | There are no MLBCs but there are plans to establish one or more of them | 2 |
|  |  | There are one or more health facilities where midwives are the only available childbirth care provider, but these are not MLBCs | 1 |
|  |  | There has never been MLBCs and there are no plans to establish them or there used to be one or more MLBCs, but currently there are none | 0 |
| 11 | **Midwife density:** Ranking by quintile for midwife density | Top quintile with highest midwife density | 5 |
|  |  | Second quintile for midwife density | 4 |
|  |  | Middle quintile for midwife density | 3 |
|  |  | Fourth quintile for midwife density | 2 |
|  |  | Bottom quintile with lowest midwife density | 1 |
|  |  | No data | 0 |
